# Supplementary material for: Validity and reliability of the XSENSOR in-shoe pressure measurement system
Source: PLoS One. 2023 Jan 17;18(1):e0277971. doi: 10.1371/journal.pone.0277971 (PMC9844836; doi:10.1371/journal.pone.0277971)
Supplement: S1 File — This file contains figures for peak pressure measures between days at each magnitude of applied pressure using the mean of the three trials for each time point (T0, T2, T10, T30). (DOCX) [file pone.0277971.s001.docx]

SUPPLEMENTARY FIGURES

Peak pressure measures for the between days comparison were assessed at each magnitude of applied pressure using the mean of the three trials for each time point (T0, T2, T10, T30). Agreement between days (Figure S1) and reproducibility between days (Figure S2) are presented for percentage difference and absolute pressure difference for both insole sizes (S4 and S10).


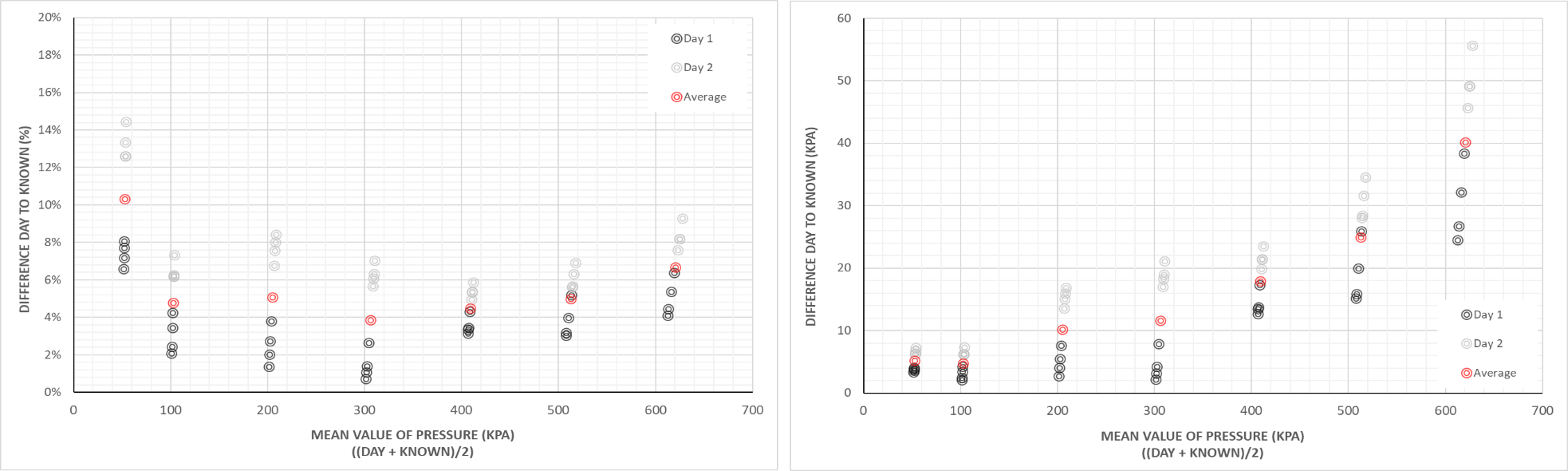

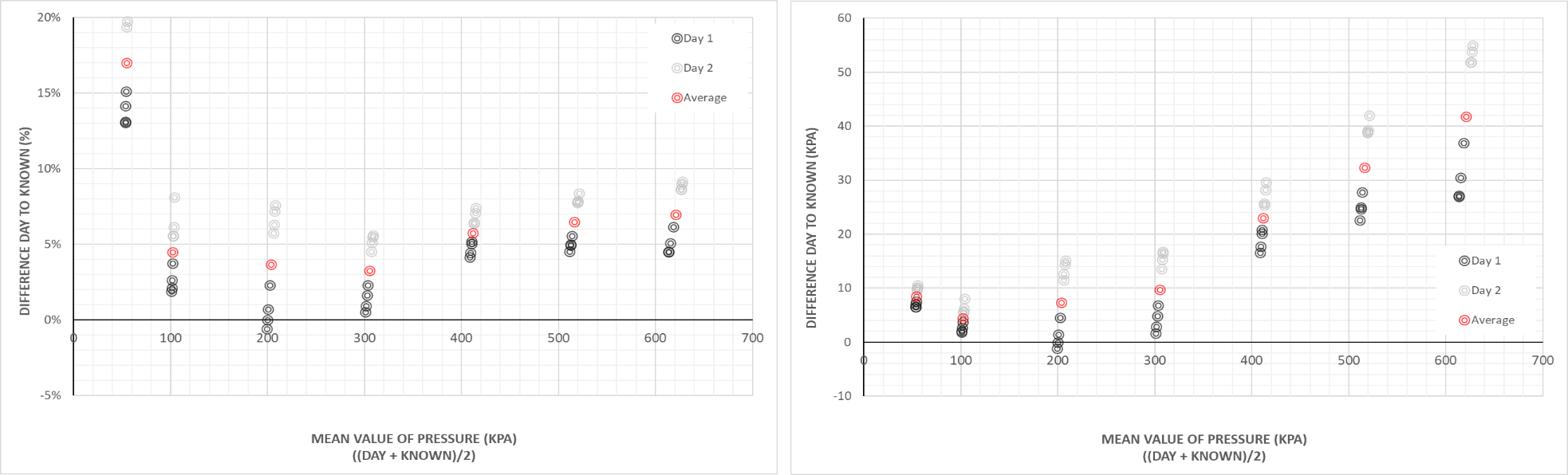


a

b

Figure X. Bland Altman plots

Figure S1. Agreement between Day one and Day two data and **target** pressure (% and kPa) using Bland-Altman plots for the S4 (a) and S10 insole (b)


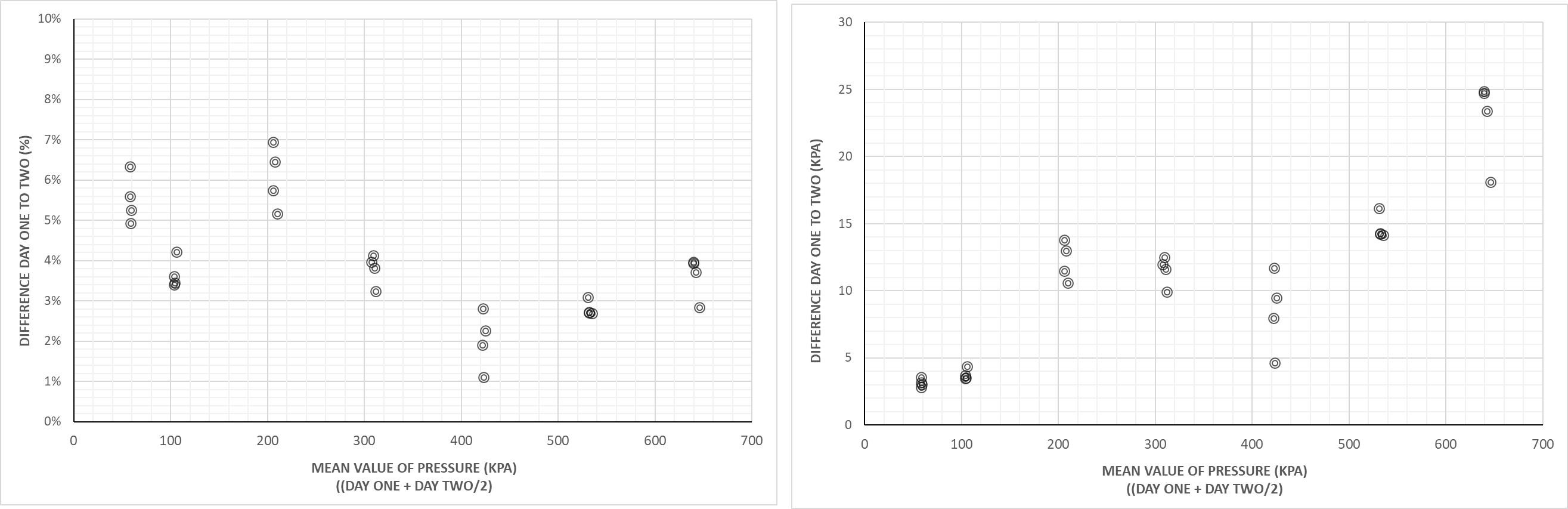


a

**
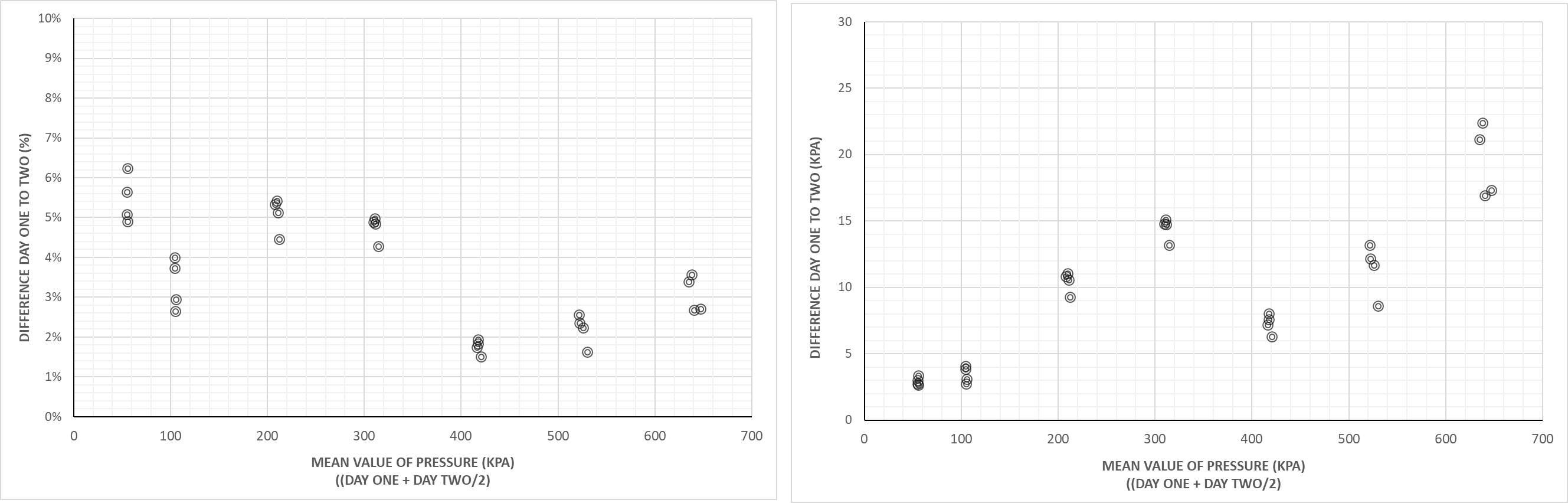
**

b

Figure S2. Reproducibility between Day one and Day two data over target pressures (% and kPa) using Bland-Altman plots for the S4 (a) and S10 insole (b)
