# Supplementary material for: Validity and reliability of the XSENSOR in-shoe pressure measurement system
Source: PLoS One. 2023 Jan 17;18(1):e0277971. doi: 10.1371/journal.pone.0277971 (PMC9844836; doi:10.1371/journal.pone.0277971)
Supplement: Attachment — Submitted filename: Reviewer comments_Round2.pdf [file pone.0277971.s005.pdf]

General comments:

I would like to congratulate the authors on the extensive revisions to the manuscript. The inclusion of Bland-Altman limits of agreement and a clinically meaningful reliability target has significantly increased the value of this manuscript to the readership. Please consider a few remaining but minor comments/questions below.

Specific comments:

Line 69: discrete

Line 74: average pressure across sensors within a discrete region

Line 96-102: I am still under the impression that this part of the introduction does not add value to the manuscript. The importance of evaluating the contact area has been demonstrated further up already. I do not understand the meaning of "Additionally, that when considering repeated measures most systems demonstrate high relationships between the two days or measures".

Line 173: intra-class correlation coefficient

Line 220-221: This sentence sounds like a contradiction. 20% > 15%

Figure 1: Is there any reason for the measurement at 100kPA showing the opposite trend of all other loading conditions? On average Figure 3 does not seem to show this opposite behavior at 100kPA.

Figure 4: (c) and (d) labels are flipped in the figure

Table 1&2: Does the correlation column represent the correlations before or after log-transformation? After transformation, the correlations should be much lower or rather non-existent. Also, there are strong negative and positive correlations (e.g. Table 1, s10, 100kPA vs. 500kPA). Do the authors have an explanation for this? I would expect a consistent trend where d2 shows higher pressures than d1 across all loading conditions?
