# Supplementary material for: Validity and reliability of the XSENSOR in-shoe pressure measurement system
Source: PLoS One. 2023 Jan 17;18(1):e0277971. doi: 10.1371/journal.pone.0277971 (PMC9844836; doi:10.1371/journal.pone.0277971)
Supplement: Attachment — Submitted filename: Response to Reviewers - PONE-D-21-30139.docx [file pone.0277971.s006.docx]

We would like to thank the reviewers for their comments on the changes made in our prior revision and careful review of the manuscript. Changes made in this revision are minor and seek to clarify the outcomes of the work.

| Reviewer #1: Thank you to the authors for their comprehensive response to the comments. I agree that the manuscript is much improved and I appreciate them taking the time to revise the content and analysis.   I have only a few minor remaining comments based on the revised version with changes highlighted | We would, again, like to thank the reviewers for their constructive first and second reviews of the paper and appreciate the opportunity to make these minor amendments. |
| --- | --- |
| Line 76: “discrete” misspelled | Changed as recommended |
| Line 97: “that” seems superfluous | Changed as recommended |
| Line 98: wording is a bit unclear; is it supposed to be “highly correlated relationships”? | This section has been reworded and moved in response to a comment from reviewer two so please see below. |
| Line 108: capitalization of international not needed | Changed as recommended |
| Line 120: missing a period. | Changed as recommended |
| Lines 142-143: inclusion of pressure gauge company redundant with information in previous paragraph. | Changed as recommended |
| Line 145: change to “were calculated” | Changed as recommended |
| Lines 224-225: I am confused by the statement that no measurements exceeded the analytical goal, when one of the 50 kPa measurements was off by 20%. | This was an error in wording and has been corrected. |
| Line 340: change to “these data” | Changed as recommended |
| Reference 11: misspelled “pressure” | This reference has been updated |
| I would like to congratulate the authors on the extensive revisions to the manuscript. The inclusion of Bland-Altman limits of agreement and a clinically meaningful reliability target has significantly increased the value of this manuscript to the readership. Please consider a few remaining but minor comments/questions below. | We would, again, like to thank the reviewers for their constructive first and second reviews of the paper and appreciate the opportunity to make these minor amendments. |
| Reviewer 2 | Line numbers match clean version |
| 1. Line 69: discrete | Changed as recommended |
| 2. Line 74: average pressure across sensors within a discrete region | Modified as recommended |
| 3. Line 96-102: I am still under the impression that this part of the introduction does not add value to the manuscript. The importance of evaluating the contact area has been demonstrated further up already. I do not understand the meaning of “Additionally, that when considering repeated measures most systems demonstrate high relationships between the two days or measures”. | Since the update of the manuscript with R1 we agree that this paragraph is repetitive with the ideas in the earlier section. We have therefore removed the majority of this section and used some of the ideas to draw a conclusion to the earlier paragraph instead of repeating the ideas. This restructure as led to the rewrite of the flagged sentence. |
| 4. Line 173: intra-class correlation coefficient | Modified as recommended |
| 5. Line 220-221: This sentence sounds like a contradiction. 20% > 15% | This was an error in wording and has been corrected. |
| 6. Figure 1: Is there any reason for the measurement at 100kPA showing the opposite trend of all other loading conditions? On average Figure 3 does not seem to show this opposite behavior at 100kPA. | The data in figure 1b refers to peak pressure values which has been added to the figure caption.  The differences identified by the reviewer are minimal in terms of value in this mid insole region, ranging from ~+5 at 100kpa to ~ to ~0 at 200kPa, but we appreciate they appear misleading in terms of making 100kpa stand out as being different to the other data.  This figure was constructed of example images from the same trial run which was intended to be representative. However, the measures selected were misleading as the 100 kPa measure was inconsistent with the others.  We have amended this and have now presented the average peak pressure difference taken across the three trial measurements for each pressure level. This now displays the individual sensor differences using the same dataset presented in figure 3. |
| 7. Figure 4: (c) and (d) labels are flipped in the figure | Figure 4 labels have been corrected |
| 8. Table 1&2: Does the correlation column represent the correlations before or after log-transformation? After transformation, the correlations should be much lower or rather non-existent. Also, there are strong negative and positive correlations (e.g. Table 1, s10, 100kPA vs. 500kPA). Do the authors have an explanation for this? I would expect a consistent trend where d2 shows higher pressures than d1 across all loading conditions? | The correlation and statistical comparison was undertaken as recommended in the literature. However these have been undertaken on only three data points for each pressure value leading to data sets of 3 for x and y. The range in X was also very low due to the X being within-pressure application. We have therefore made the decision to exclude the correlations from this table as they are misleading. Instead, we have added the correlation across the target pressure values for day one versus day two, which is more consistent with the implementation of this method in the literature, spanning a range of multiple x and y values, therefore expecting heteroscedastic error. As opposed to the x value only varying with systematic error.  In line with this response, the following changes have been made to the manuscript:  - the method for the statistical comparisons updated  - the columns relating to correlation from Table 1 (average), Table 2 (peak) have been removed  - we have added the data for t-test and correlation across applied pressure values into the written text within the result section and refer to this in the discussion. |
