# Supplementary material for: Validity and reliability of the XSENSOR in-shoe pressure measurement system
Source: PLoS One. 2023 Jan 17;18(1):e0277971. doi: 10.1371/journal.pone.0277971 (PMC9844836; doi:10.1371/journal.pone.0277971)
Supplement: Attachment — Submitted filename: Reviewer comments.pdf [file pone.0277971.s003.pdf]

General comments:

This manuscript investigates the validity and day-to-day repeatability of an XSensor in-shoe pressure-sensing insoles during standardized loading protocols in comparison to known pressures and contact areas.

Overall, I agree with the authors on the importance for independent research groups to confirm the validity and reliability of commercial measurement systems. I also get the impression that the measurements were carried out carefully. In an original research article, however, the generated data should be presented and discussed in a way that provides value to other researchers. In my opinion, the submitted manuscript is lacking a clear structure, the investigated outcome variables are not well justified, and there are no criteria for what constitutes sufficient validity and reliability. My main critical points are:

1) The outcome variables are not well prepared and justified in the introduction. The authors talk about mean and peak pressure over varying duration, contact areas at different pressures, creep, insole consistency, etc.. It is unclear, why each variable is of interested and in which context each variable is relevant. The aim statement should clearly state the variables that the authors plan to use to determine the insoles' validity and repeatability. Then, the variables should be described in the methods. Particularly, the authors should avoid to report results that were not prepared in the methods (creep, insole consistency).

2) What constitutes sufficient validity and repeatability? The authors talk about various applications of pressure-sensing insoles, e.g. running biomechanics [2] or in patients with neuromotor disorders [3-5]. Comparing pressures between slightly modified running shoes during running OR comparing pressures between healthy vs. diseased patients during standing or walking will lead to very different requirements of validity and repeatability for a pressure-sensing system. Therefore, the authors cannot make a sweeping statement in the abstract to say that "the Xsensor system is appropriate for clinical assessment that require multiple assessments".

   Therefore, the criteria that indicate sufficient validity and repeatability need to be developed carefully in the context of a certain research area and are only relevant in this context. Those criteria should not only be based on relative measures (e.g. relative error or ICC). Such measures certainly have their strengths but also weaknesses (e.g. Koo, Terry K., and Mae Y. Li. 2016. "A Guideline of Selecting and Reporting Intraclass Correlation Coefficients for Reliability Research." *Journal of Chiropractic Medicine* 15 (2): 155–63.)
   The authors should consider including absolute measures such as Bland-Altman limits of agreement (see Atkinson, G., and A. M. Nevill. 1998. "Statistical Methods for Assessing Measurement Error (reliability) in Variables Relevant to Sports Medicine." *Sports Medicine* 26 (4): 217–38.). Limits of agreement would provide an intuitive way of determining whether the measurement error grows with increasing pressure or measurement duration.

   Finally, the authors should come up with clinically relevant or meaningful changes in pressure in absolute physical units (or maybe relative to body weight) that occur in a certain research context and use those values to build a framework for validity and repeatability.

3) The authors criticize previous studies for their limited external validity. In this experiment however, there is no information on the loading rate. I assume that the pressure was built up slowly and was not comparable to a pressure profile to running or walking. Is there anyway

for your system to simulate a dynamic loading profile? Currently, I would argue that the results of this manuscript are relevant for standing only.

Specific comments:

Line 72-73: ICCs of around 0.5 are not usually considered to show “high intra class correlation”.

Line 73-81: The relevance of previous findings on the Tekscan system is unclear for this study on the XSensor system. It would seem more relevant to explain if some measurement systems have a larger error than clinically relevant underfoot pressure changes in clinical populations.

Line 95: It is unclear how the “suitability for clinical and biomechanical assessment” is defined. There should be a-prior standards about the validity and repeatability for their use in clinical research and biomechanical research.

Line 99: When looking at the XSensor webpage (https://xsensor.com/), the available systems are “Intelligent Insoles Clinical” and “Intelligent Insoles Pro”. Please explain how the XSensor X4 system relates to these two available systems.

Line 115: How long were the measurements?

Line 117: Where exactly were those sensels and how many were excluded?

Line 118: sensel underlined?

Line 125: Please briefly describe the testing device to generate pressure during the regional protocol. How accurate is this device?

Line 129: How long were the measurements in the regional protocol?

Line 130: Where was the regional pressure applied?

Line 138: This is the first time that a between-day study design is mentioned. This must be mentioned in the aims of this study and at the beginning of the methods. In general, while it is appreciated that the previously developed protocol is cited [reference 18], the most important aspects of this protocol must still be mentioned. Like how many days passed between the measurements, etc.? Why were the durations 0,2, 10, 30 seconds selected? What is a duration of 0 seconds anyway? How exactly were relative and absolute errors calculated (e.g. the ones in Table 2)?

Lines 134-144: This section is difficult to follow. For example: What happened to the different pressures in the regional protocol (50, 110, 200 kPa)? Why were peak pressures investigated? Is “load” equal to pressure? Why did the authors only investigate ICCs as a relative measure of reliability? In my opinion, absolute measures of repeatability and validity such as Bland-Altman limits of agreement would be much more intuitive in their interpretation and it would be easier to observe changes in agreement with the known measures as a function of held duration or applied pressure. This section needs to be structured better and the analysis approach should be justified.

Line 143: Please explain exactly what type of ICC model was used and how the individual values were combined into the respective results. Different ICC models can lead to different results given the same data.

Figure 1: Please add a color scale so the reader can assess changes in pressure. Please indicate where in Figure 1a the regional pressures were applied.

Line 154: Why is there no analysis of the measured pressures in the regional protocol? This type of regional loading may affect the insole's validity in measuring the correct mean and peak pressure.

Table 1: There is no description of this analysis in the methods.

Lines 160-167: It is unclear where those values come from? (e.g. S10 (23.09±15.92kPa; 9%)). Are those averages?

Line 164-165: There has been no mention of a time-dependent analysis of peak pressure in the methods section.
